# Bibliometric analysis of the *S24-7* family and its association with health

**DOI:** 10.3389/fmicb.2025.1571883

**Published:** 2025-05-08

**Authors:** Fangmei Gao, Fenfen Wang, Dandan Wang, Guankui Du, Fangfang Gao

**Affiliations:** ^1^Department of Breast Surgery, The First Affiliated Hospital of Hainan Medical University, Haikou, China; ^2^Key Laboratory of Tropical Translational Medicine of Ministry of Education, School of Basic Medicine and Life Sciences, Hainan Medical University, Haikou, China; ^3^The Second People’s Hospital of Dongfang City, Dongfang, China; ^4^Qunying Health Center of Lingshui Li Autonomous County, Lingshui, China; ^5^Yongming Health Center of Ledong Li Autonomous County, Ledong, China; ^6^Biotechnology and Biochemistry Laboratory, Hainan Medical University, Haikou, China; ^7^Department of Biochemistry and Molecular Biology, Hainan Medical University, Haikou, China

**Keywords:** *S24-7*, gut microbiota, research hotspots, bibliometric analysis, health

## Abstract

A burgeoning corpus of evidence indicates that *S24-7* is integral to human health, with links to obesity, inflammation, metabolism, and dietary interactions. In the present study, we conducted a comprehensive review of the *S24-7* literature from the past 10 years, augmented by an evaluation of research trends using both quantitative and qualitative approaches. From the Web of Science (WoS) database, we retrieved 903 research articles and four review articles pertaining to *S24-7*, also known as Muribaculaceae, that were published between January 1, 2014, and January 1, 2024. Employing software tools such as R, Biblioshiny, VOSviewer, the Bibliometric Analysis Platform, and Pajek, we performed visual mapping and correlation analyses on the collected documents. Our analysis revealed China and the United States as the leading publishers in the field of *S24-7* research. The top three academic journals for *S24-7* family research are Food and Function, Frontiers in Microbiology, and Nutrition. Among individual contributors, Zhang Y stands out with 31 publications and an h-index of 13, representing 3.42% of the 907 articles analyzed. Jiangnan University leads in institutional output with 46 publications. Keyword analysis underscores that *S24-7* research is concentrated on examining the family’s associations with obesity, inflammation, metabolism, and diet. This study highlights notable contributions from various countries, institutions, journals, and researchers, shedding light on the influence of the *S24-7* family on human health. It serves to inform future research directions and clinical applications concerning the *S24-7* flora.

## 1 Introduction

The human gut microbiota is teeming with billions of microbes that play a vital role in sustaining host health (Gomaa, 2020; Cryan and O'Mahony, 2011). Within this complex ecosystem, the *S24-7* family, a prominent constituent of the Mycobacteriaceae phylum, holds significant relevance to human well-being. Saltzman and colleagues initially identified this family as part of the mouse intestinal microbiota (Seedorf et al., 2014). Later, Sidov et al. bestowed upon it the designation “*S24-7*” (Salzman et al., 2002). Ormerod et al. conducted a seminal study, sequencing the genomes of 30 *S24-7* members and positioning them within the novel candidate family Candidatus Homeothermaceae (Ormerod et al., 2016). Genome analysis revealed that *S24-7* spp. are functionally distinct from neighboring families and versatile with respect to complex carbohydrate degradation (Lagkouvardos et al., 2019). Bacteria belonging to the *S24-7* constitute a predominant element of the mammalian gut microbiota.

Endowed with an expansive repertoire of carbohydrate-active enzymes, *S24-7* members are adept at fermenting a diverse array of carbohydrates within the digestive tract. Moreover, *S24-7* operates independently of T-cell-mediated immunity, fostering the production of Immunoglobulin A (IgA) (Bunker et al., 2015). This family also boasts an abundance of genes encoding enzymes that dismantle plant cell walls. Carbohydrate-active enzymes account for approximately 6% of the *S24-7* coding sequence (Ormerod et al., 2016), with the GH13 family of glycoside hydrolases being particularly prevalent, chiefly comprising α-amylases. Trailing behind are the GH43 family, predominated by xylosidases and arabinosides. Subsequently, a gene pair analogous to susCD, integral to *S24-7*, was pinpointed at the genetic level, and susC/susD-like gene pairs typically form part of PUL clusters (Xu et al., 2003; Martens et al., 2009). These loci are defined by the structural components of the starch utilization system (Sus), which orchestrates the binding of starch and malt oligosaccharides to SusD and their translocation to the periplasmic space via SusC (Reeves et al., 1996; Reeves et al., 1997). Through the Sus system, the *S24-7* exerts regulatory control over polysaccharide catabolism. Additionally, research has revealed that *S24-7* exhibits a pronounced affinity for whole wheat grains (Garcia-Mazcorro et al., 2016). Furthermore, this family possesses a suite of genes encoding the electron transport chain and ATP-synthesizing enzymes, thereby contributing significantly to the acceleration of energy metabolism (Ormerod et al., 2016). Collectively, these attributes underscore *S24-7*’s pivotal role in intestinal functionality and health. Consequently, there is a compelling imperative to delve into the dynamics of the *S24-7* in relation to health trends.

Bibliometrics, a discipline harnessing both qualitative and quantitative analytical techniques (Ninkov et al., 2022), employs the examination of recurrent keywords in extant literature to illuminate prevailing themes and burgeoning trends across the entire spectrum of research domains. Presently, there exists a dearth of bibliometric investigations concerning *S24-7*. Our endeavor aims to bridge this gap by elucidating the current research foci and forecasting prospective avenues for advancement in this area. This initiative is intended to furnish a foundation for more rigorous and focused explorations into the intricacies of gut microbiota, which in turn may yield novel diagnostic markers and innovative therapeutic strategies for a myriad of diseases.

## 2 Materials and methods

This study undertook a comprehensive review of the literature pertaining to *S24-7*, sourced from the Web of Science (WoS) database. The search spanned from January 1, 2014, to January 1, 2024, employing search terms inclusive of “*S24-7*” or “Muribaculaceae.” Original research articles and review papers were exclusively selected, while other forms of literature, including those from conferences and additional reviews, were excluded. The final dataset encompassed 907 articles relevant to the specified topic. For data analysis, a combination of statistical software R, its Biblioshiny extension, VOSviewer, the Bibliometric Analysis Platform, and Pajek were utilized to generate tabular datasets and visual representations. The R-based Biblioshiny application, accessible at https://bibliometrix.org/, facilitated the creation of bibliometric visualizations, including maps illustrating international collaborations, author productivity, and the corresponding impact factors (Hasan et al., 2023). VOSviewer, a web-based tool, specializes in the generation of maps based on bibliographic or textual data (van Eck and Waltman, 2010). It visually represents the co-occurrence of keywords, which is subsequently refined and optimized through the use of Pajek software. Additionally, the bibliometric analysis platform offered a detailed overview of the annual publication output per country.

According to the statistical results of the WOS database, we used R Studio to generate the Biblioshiny application for related data such as institutions, journals, and authors. Additionally, a map of the number of publications by institutions and journals, the number of authors’ literature published and cited, and the distribution of maps using RStudio were generated. The bar charts of article publications by country for each year and country collaboration maps using the bibliometric analysis platform was generated. The keywords were analyzed for hotspots using VOSviewer, and then the data were saved as Pajek files and imported into Pajek software to draw a clearer cluster analysis of the hotspot words.

## 3 Results

### 3.1 Analysis of nations and institutions

**Figure 1:**
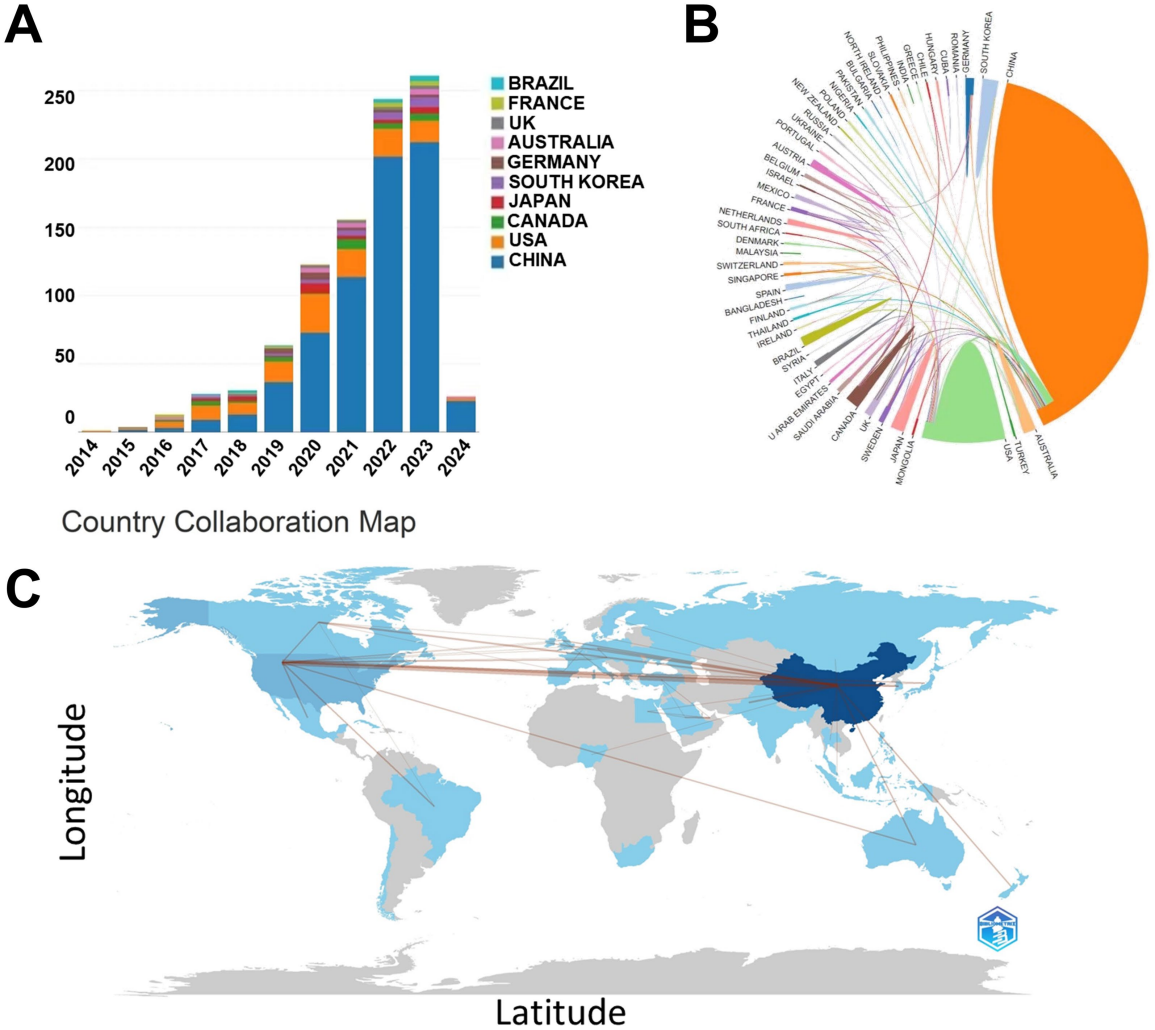
Article releases in the field by country. **(A)** Number of article releases in the field by country in different years. **(B)** The cooperation and **(C)** partnerships of countries in the field of *S24-7* from 2014 to 2024.

Over the course of the 10-year investigation, publications pertaining to the *S24-7* appeared across 53 countries. China emerged as the foremost contributor with 688 articles, accounting for 75.85% of the total 907 papers, closely followed by the United States with 128 papers (14.11%). Canada contributed 25 articles (2.76%), Japan 24 (2.65%), and South Korea 23 (2.54%) (Figure 1A). The collaborative network visualization map illustrated both the quantity of publications—with darker shades denoting higher numbers—and the connections indicating collaborative ties (Figures 1B,C).

**Figure 2:**
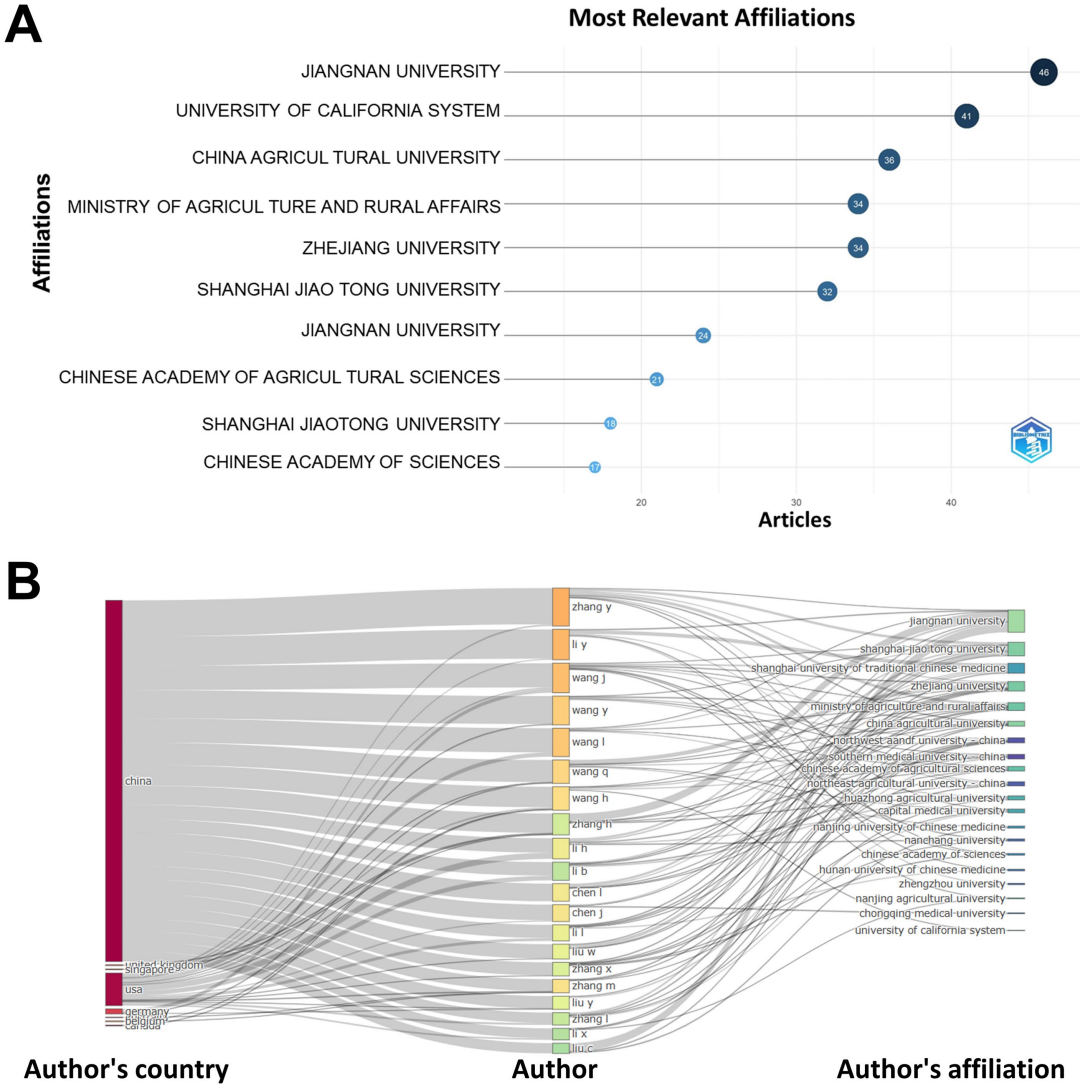
Institutional literature releases in the field. **(A)** Number of article releases in the field by institution. **(B)** Map of collaborative networks of countries, institutions and authors.

In terms of institutional productivity, Jiangnan University was the most prolific with 46 publications, trailed by the University of California System with 41 and China Agricultural University with 36. Jiangnan University not only led in the number of publications but also stood out for its high degree of collaboration (Figure 2A). Moreover, Figure 2B presented a connectivity diagram showcasing the top 10 countries, institutions, and authors by volume of published work, reflecting robust inter-agency and cross-institutional cooperative efforts.

### 3.2 Analysis of journals

**Figure 3:**
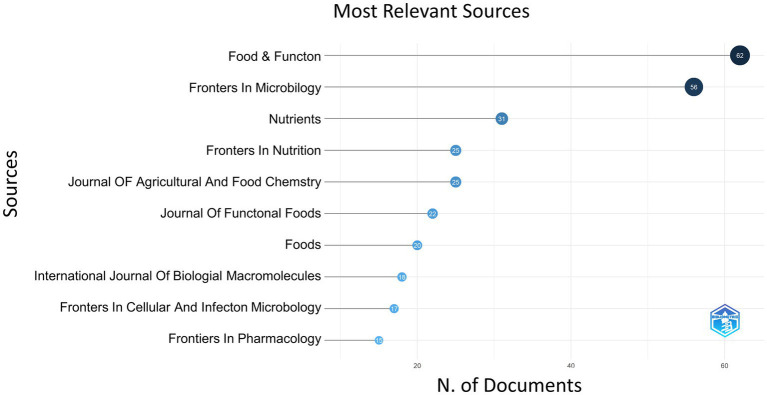
Ranking of journal literature releases in the field.

Publications on the *S24-7* appeared in 299 distinct scientific journals, with Food and Function leading the list with 62 articles, representing 6.84% of the total. Frontiers in Microbiology came in second with 56 articles (6.17%); Nutrients published 31 articles (3.42%); Frontiers in Nutrition and Journal of Agricultural and Food Chemistry both contributed 25 articles each, accounting for 2.76%. Figure 3 detailed the top 10 journals ranked by the number of published papers. The average impact factor for the top five journals was approximately 6, and notably, the majority were categorized in Q1, highlighting the high caliber of the publishing venues.

### 3.3 Analysis of authors and references

**Figure 4:**
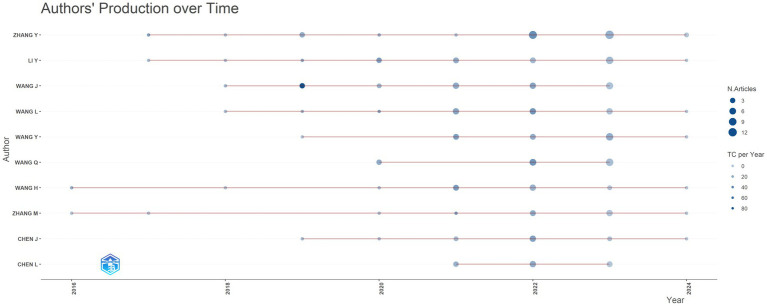
Author literature releases and citations in this field.

Zhang stood out as the most prolific author in the *S24-7* research domain, having authored 31 papers (3.42% of 907 total publications) (Figure 4). Wang followed closely behind with 23 publications (2.54%). Both individuals boast relatively high h-indices of 13 and 12, respectively. Notably, they both hail from China and have engaged in collaborations with numerous other authors and organizations.

### 3.4 Analysis of keywords

**Figure 5:**
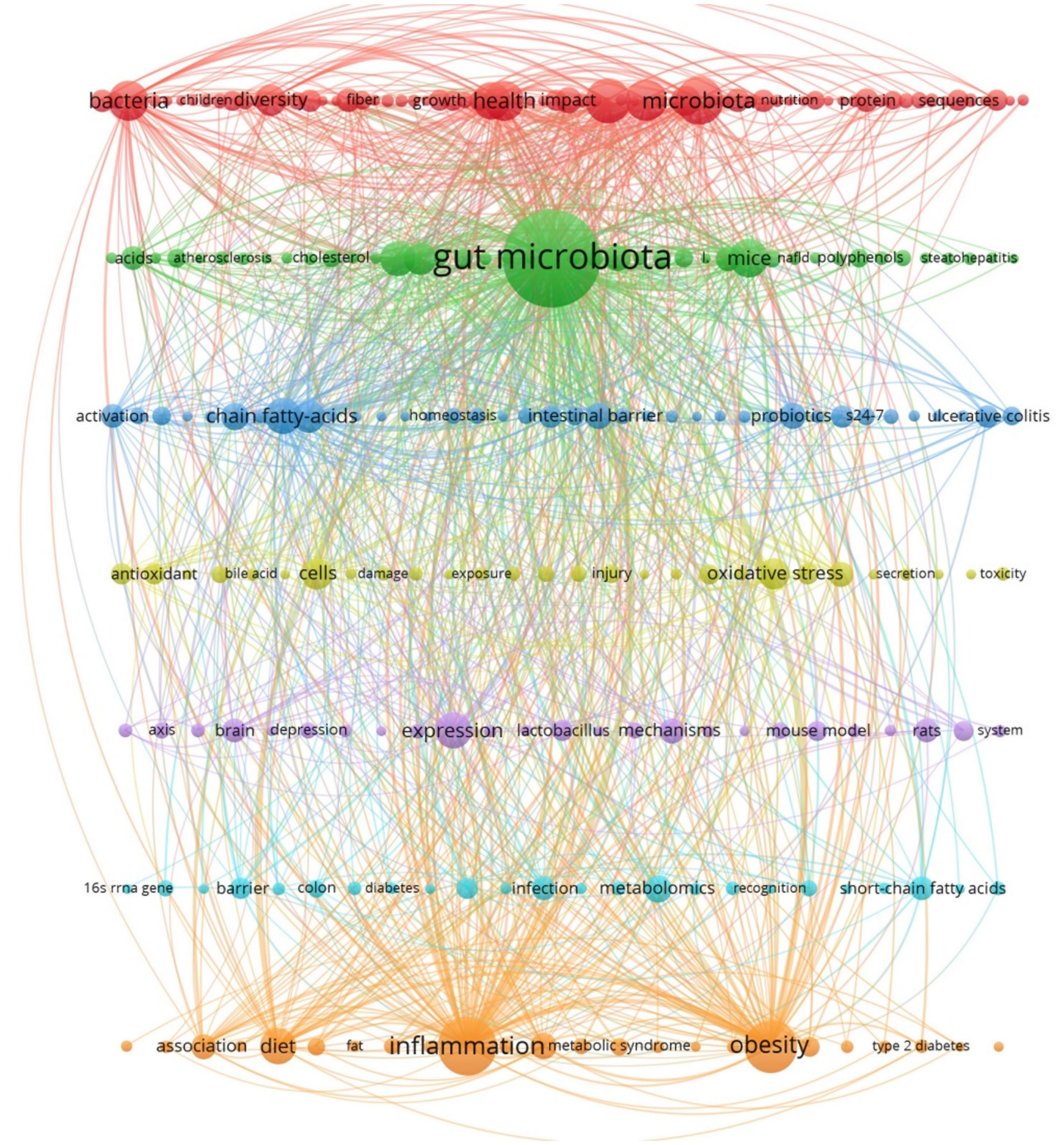
Keyword co-occurrence map for this domain.

High-frequency keywords served as effective indicators of research focal points and trends. Through the use of VOSviewer, 3,848 keywords were extracted from a dataset of 907 articles, with those appearing at least six times designated as high-frequency keywords, totaling 253 (Figure 5). Common themes among these keywords included gut microbiota, inflammation, obesity, metabolism, health, and diet. Research trends suggested a focus on the implications of *S24-7* abundance variations for human health, with specific interests in its role in obesity, inflammation, and metabolism, as well as the impact of dietary patterns on the prevalence of *S24-7*.

## 4 Discussion

Numerous studies have underscored the critical role of the gut microbiome in human health (Milani et al., 2017). The *S24-7* is closely tied to obesity, inflammation, metabolism, and dietary composition in mammals (Li et al., 2019). It is anticipated to become a key biomarker for diagnosing human diseases and guiding therapeutic strategies, thereby enhancing our understanding of naturally occurring therapeutic agents and disease prevention measures.

To delineate the research hotspots and trends within the *S24-7* family, a bibliometric analysis was conducted on relevant articles spanning from 2014 to 2024. This yielded 907 *S24-7*-associated papers, with China being the leading contributor, followed by the United States. Among institutions, Jiangnan University led with 46 publications, closely followed by the University of California system with 41 and China Agricultural University with 36. These findings indicate a collaborative effort among the mentioned countries and organizations. The analysis revealed that within the past decade, a significant number of researchers and institutions have concentrated on examining the link between the *S24-7* and human health. Specifically, there has been a surge in studies exploring the correlation between gut microbiota distribution and obesity, inflammation, and metabolism (Li et al., 2019; Lin et al., 2020; Xu et al., 2020). Moreover, experimental research has begun to investigate the impact of dietary structures on the abundance of the *S24-7* (Zhao et al., 2019). The thematic development and research trends of the *S24-7* have been elucidated through keyword hotspot distribution maps and development maps. Preeminent themes include the gut microbiota, obesity, inflammation, metabolism, and diet. Leveraging advanced technological capabilities and the establishment of sophisticated mammalian experimental models, future research is poised to delve deeper into the *S24-7* family’s connection with human health.

Over the past decade, a growing number of scholars and countries have expressed increasing concern about the relationship between the *S24-7* and obesity. As a significant public health issue, obesity has escalated into an epidemic (Hill et al., 2003). Raoult et al. posit that obesity is multifactorially influenced by environmental, genetic, neurological, and endocrine elements (Raoult, 2008). Alterations in the human gut microbiome are also implicated in obesity. Previous research indicates that obesity correlates with an expansion of thick-walled bacterial phyla and a reduction in bacillus-like phyla (Turnbaugh et al., 2006; Bäckhed et al., 2004). *S24-7* exhibits a strong association with obesity, and a high-fat diet can markedly influence the microbiome composition, characterized by elevations in Sclerotinia, Proteobacteria, and Verrucomicrobia, along with diminished levels of mycobacterium-like organisms (the *S24-7* family) and Candida arthritis (Tomas et al., 2016). Mechanistic investigations into the link between *S24-7* and obesity often suggest that elevated levels of bacillus-like *S24-7* contribute to increased butyric acid production, which subsequently impacts metabolism (Rose et al., 2018). Butyric acid plays a crucial role in regulating mitochondrial function, including enhancing oxidative phosphorylation and β-oxidation, thereby optimizing cellular metabolic processes. Furthermore, butyric acid stimulates genes involved in fat oxidation, attenuates glycolysis, and boosts fat metabolism (Mollica et al., 2017; Valvassori et al., 2013). Additional studies demonstrate that butyric acid facilitates mitochondrial fission, thereby augmenting NADH production, intensifying the tricarboxylic acid cycle, and ultimately fostering nutrient breakdown while curtailing metabolite accumulation (Mollica et al., 2017; Valvassori et al., 2013). In a murine model of obesity induced by a high-fat diet, Henagan et al. observed similar outcomes, noting that butyric acid amplifies mitochondrial oxidative phosphorylation, upregulates the expression of fatty acid oxidase and uncoupling protein (UCP) 69, and initiates the nuclear coding of mitochondrial genes, including peroxisome proliferator-activated receptor (PGC1α), culminating in enhanced β-oxidation and improved insulin sensitivity (Henagan et al., 2015). Butyric acid also encourages the conversion of white adipose tissue (WAT) into brown adipose tissue (BAT), thereby increasing host energy expenditure (Li et al., 2019; Li et al., 2018; Montanari et al., 2017; Kajimura and Saito, 2014). Moreover, emerging evidence suggests that maternal obesity may disrupt placental oxidation and inflammatory responses by altering the gut microbiota composition (Hu et al., 2021). Future research endeavors may illuminate potential targets for elucidating the interplay between the *S24-7* and obesity, potentially leading to novel strategies for obesity management.

A burgeoning body of research has established connections between the *S24-7* and inflammation, particularly within the gastrointestinal tract. The *S24-7* has been implicated in the pathogenesis of inflammatory bowel disease (IBD), with notable alterations in the microbial ecosystem observed in ulcerative colitis (UC) characterized by an elevated Bacillus thickeniensis to Bacillus anthropophilus ratio (Meisel et al., 2017). Interleukin-15 (IL-15), an inflammatory cytokine that perturbs gut microbiota equilibrium, has been shown to diminish *S24-7* abundance and butyric acid levels in both the gut and feces of mice, precipitating intestinal inflammation (Zou et al., 2020). Conversely, Li-Zhong Tang’s research demonstrated that enhancing the *S24-7* in mice could modulate the MAPK pathway, restructure the gut microbiota, reduce inflammatory cell infiltration, attenuate tumor necrosis factor activity, and dampen the inflammatory response (Zou et al., 2020). Certain bioactive compounds, such as *Ficus carica* polysaccharide (FCPS), human milk oligosaccharides (HMOs), components derived from legumes, and cinnamon essential oil obtained from fig fruits, have been identified as capable of elevating *S24-7* levels in mice. These substances play a pivotal role in regulating inflammatory responses, maintaining intestinal endothelial integrity, and producing bioactive molecules that promote intestinal health, including phenolic metabolites and short-chain fatty acids (SCFAs). Their application holds promise for the prevention and treatment of IBD (Zou et al., 2020; Monk et al., 2016; Li et al., 2020). Additionally, supplementation with probiotics like Lactobacillus royale has been shown to diversify the gut microbiome, increase *S24-7* abundance, enrich bacterial motility proteins, and ameliorate mucositis induced by the chemotherapeutic agent 5-FU, offering a novel approach to mitigating chemotherapy-induced bacterial injury (Yeung et al., 2020). Conversely, a low-fiber diet has been associated with reduced *S24-7* levels in mouse models and is known to disturb the gut microbiome, potentially triggering underlying inflammatory processes that contribute to the exacerbation of inflammatory states (Makki et al., 2018). It has been shown that atherosclerosis and systemic inflammatory response were also reduced in the group using SGLT2 compared to the control group, with higher levels of *S24-7* in the feces (Hao et al., 2023). Collectively, these findings underscore the intimate relationship between the *S24-7* and intestinal inflammation, positioning it as a current focal point of research interest and laying the groundwork for future investigative efforts.

Gut microbes engage in co-metabolic processes with their hosts, furnishing them with essential enzymes that they cannot produce autonomously (Tremaroli and Bäckhed, 2012). Research has indicated that fluctuations in the abundance of the *S24-7* correlate with lipid metabolism. Specifically, Chai Gui Tang and its principal constituents have been shown to mitigate spontaneous hypertension through the dual regulation of lipid metabolism and the maintenance of gut microbiota equilibrium, thereby contributing to the attenuation of this condition (Zhu et al., 2023). Expanding on this, *Bacillus amyloliquefaciens* SC06 has been identified as a potent modulator of mammalian obesity. It achieves this by reshaping the composition of the gut microbiota and modulating bile acid metabolism via the FXR signaling pathway (Zeng et al., 2022). Additionally, enzymes produced by the *S24-7* contribute to the breakdown of bile acids, which in turn influences bile acid metabolism, alters hepatic gene expression, and governs liver regeneration (Liu et al., 2016). An increased ratio of Bacillus thickeniensis to Bacillus anthropophilus has been linked to the progression of type 2 diabetes mellitus (T2DM) in mouse models (Goodman et al., 2011; Pedersen et al., 2016). Conversely, the presence of the *S24-7* appears to confer protection against obesity-induced insulin resistance (Louis and Flint, 2009). Given the intricate relationships between the *S24-7* and various facets of metabolism, it is evident that targeted research on this microbial entity could serve as a valuable reference for future drug development initiatives aimed at metabolic disorders. By delving deeper into the mechanisms underpinning the interactions between the *S24-7* and host metabolism, scientists may uncover new avenues for therapeutic intervention in metabolism-related diseases.

Dietary components precipitate shifts in the levels of *S24-7* within the gut microbiota (Kurup et al., 2021; Wang et al., 2020). Notably, under conditions of a low-protein diet, the abundance of *S24-7* experiences a substantial increase (Shen et al., 2016). The incorporation of pectin has been observed to elevate the relative abundance of the *S24-7* group relative to a sugar-free diet control group (Wang et al., 2020). Furthermore, preliminary studies suggest that a combination of wasabi stems and rhizomes may exert a beneficial influence on cardiovascular health by modulating the abundance of the *S24-7* population. Investigating the metabolic impact of two cinnamon bark extracts rich in polyphenols and grape pomace on mice fed a high-fat diet for 8 weeks, Van Hul M et al. reported that the introduction of sugars diminished the prevalence of *S24-7*, whereas a higher intake of fermentable fiber had the opposite effect, augmenting *S24-7* levels (Van Hul et al., 2018). In synthesis, the dynamic between *S24-7* and dietary intake may be partially attributable to its responsiveness to dietary fiber and protein. Given that the human genome lacks a comprehensive array of carbohydrate-active enzymes, dietary fiber escapes digestion in the stomach and small intestine and instead undergoes fermentation in the colon by the resident gut microbiota (Angelakis et al., 2012). While much of the current understanding regarding the dietary determinants of *S24-7* abundance stems from mouse model studies, the translation of these insights into strategies for regulating *S24-7* populations in humans for health benefits requires further elucidation.

### 4.1 Strengths and limitations

The bibliometric analysis and visualization methodology employed in this investigation to gage the volume and quality of research concerning the *S24-7* inherently possess certain constraints. Initially, the analysis was confined to a solitary database for literature search, which may have limited the scope of the findings. Additionally, the search was restricted to article titles, potentially omitting relevant works that might not have been indexed under the specified search terms. Despite these caveats, our study presents the inaugural visual bibliometric analysis within this domain. Our findings offer an empirical overview of the foremost nations, institutions, publishing outlets, researchers, and keywords that have shaped the field over the past 10 years. This data paints a vivid portrait of the current landscape and prevailing trends in *S24-7* research. Our analysis contributes meaningfully to the characterization of the *S24-7* and sets a foundation for future investigations. Even with its acknowledged limitations, the insights gained from this study can catalyze a more profound understanding of the *S24-7* within the scientific community.

## 5 Conclusion

The current research hotspot and trend revolve around the intricate associations between the *S24-7* and human conditions such as obesity, inflammation, metabolism, and diet. With deepening insights into the *S24-7*, the landscape of disease diagnostics and targeted therapies is continuously reshaped. This progression not only enhances our understanding of naturally occurring therapeutic agents but also paves the way for innovative strategies in disease prevention. In light of these developments, the demand for sustained research efforts and conclusive findings is paramount to propel the field forward.

## Data Availability

The original contributions presented in the study are included in the article/supplementary material, further inquiries can be directed to the corresponding authors.
